# Evaluation of Effect of Different Insertion Speeds and Torques on Implant Placement Condition and Removal Torque in Polyurethane Dense D1 Bone Model

**DOI:** 10.3390/polym16101361

**Published:** 2024-05-10

**Authors:** Zeynep Dilan Orhan, Levent Ciğerim

**Affiliations:** Department of Oral and Maxillofacial Surgery, Faculty of Dentistry, Van Yuzuncu Yil University, Van 65090, Turkey; zeynepdilanorhan@yyu.edu.tr

**Keywords:** polyurethane model, polyurethane foam, polyurethane plate, dental implant, insertion torque, removal torque, implant placement condition

## Abstract

The aim of this study was to evaluate the effect of two different insertion speeds at eight different insertion torque values ranging from 25 to 60 during implantation in a dense polyurethane (PU) D1 bone model on the placement condition and removal torque of dental implants. In this study, 50 pcf single-layer PU plates were used. In the study, a total of 320 implant sockets were divided into two groups, Group 1 (30 rpm) and Group 2 (50 rpm), in terms of insertion speed. Group 1 and Group 2 were divided into eight subgroups with 25, 30, 35, 40, 45, 50, 55 and 60 torques. There were 20 implant sockets in each subgroup. During the implantations, the implant placement condition and removal torque values were assessed. There was a statistically significant difference between the 30 and 50 rpm groups in terms of overall implant placement condition (*p* < 0.01). It was found that the removal torque values at 50 rpm were statistically significantly higher than those at 30 rpm (*p* < 0.01). This study showed that in dense D1 bone, the minimum parameters at which all implants could be placed at the bone level were 50 torque at 30 rpm and 40 torque at 50 rpm.

## 1. Introduction

Dental implants have been widely used over the past 30 years because of their ability to replace missing teeth and restore the ultimate goal of modern dentistry, namely to reestablish the patient’s function, speech, health and aesthetics, regardless of stomatognathic atrophy, disease or injury [1]. It provides an advanced therapy that closely resembles the look, feel and function of natural teeth, including speech and chewing. Unlike removable dentures, implant-supported dentures do not move during function. This helps to maintain the contour and attractiveness of the face by reducing bone loss and changes to nearby healthy teeth [2]. Craniofacial reconstructions, skeletal anchors and orthodontic appliances are also stabilised by dental implants [3]. Therefore, dental implants are currently a very prominent alternative and have allowed significant development in dental, oral and maxillofacial surgery due to their high success rate, predictability and reliability of treatment and relatively minimal complications [4]. Research has shown that although many design aspects play an important role, an individual’s bone quality is a major component in predicting the effectiveness of dental implants. In terms of patient characteristics and health status, the majority of dental implants in healthy individuals have a success rate of between 90 and 95% after ten years [5,6].

Several factors have been identified as critical to implant success and stability [7]. There are two types of implant stability. It is a biomechanical phenomenon involving the quality and quantity of bone at the implant site required for implant osseointegration. Secondary stability follows the healing period and corresponds to primary stability, improving as new bone develops and matures at the interface. The patient’s medical condition, bone quality, surgical technique, biomaterial composition, implant width, length, and geometry, biomechanical considerations and surface characteristics are all important considerations in dental implant planning in order to improve osseointegration and, ultimately, the long-term success of the implant [8,9]. Bone density is an important factor to consider when assessing implant stability. Failure rates are generally attributed to inadequate bone quantity and/or quality, resulting in inadequate fixation [10]. Dental implants placed in the mandible are more likely to survive than those placed in the maxilla, particularly in the posterior maxilla [11]. Clinicians generally believe that bone quality is the main reason for the difference in survival rates between the upper and lower jaw [12]. Implants inserted in type IV bone may have a higher failure risk, according to numerous studies in the literature. Researchers have also documented positive results from implants inserted into type I, II, and III bone [13].

Various materials have been used in the literature for in vitro bone modelling. Human and animal cadavers and polymers are commonly used [14,15,16]. Human cadavers and animal models have similar properties to natural tissues but have disadvantages such as biosafety, safe transport and storage costs. Polyurethane (PU) is a family of polymers with diverse properties and applications that are all based on the exothermic reaction of organic polyisocyanates with polyols. It is used in many different medical fields, including vascular and orthopaedic, due to its unique mechanical properties and biocompatibility [17,18,19]. The American Society for Testing and Materials has accepted PU sheets as an alternative material for the biomechanical testing and evaluation of dental implants. PU sheets do not mimic human bone structure but have mechanical properties similar to bone tissue. Their mechanical properties allow the standardisation of procedures by eliminating existing anatomical and structural differences in bone [20,21]. PU foam sheets have been identified as the most suitable material for in vitro use to simulate bone tissue and different densities to compare the stability of dental implants and bone screws [22]. However, PU is reliable, easy to use and does not require any special treatment [23].

Bone density at the implant site influences the optimal loading time, implant design, and treatment strategy [24,25]. There is a linear correlation between primary stability levels and bone density [13,26]. These studies have used different techniques to assess bone density and primary stability. Insertion torque (IT) measurements are a widely used technique for the objective assessment of primary stability [27]. The ideal IT has been the subject of numerous studies that have been published in the literature [12,28]. Although there appears to be no consensus in these studies, torque values between 25 and 70 are recommended as implant IT. In addition, some implant manufacturers have established maximum torque values to prevent structural damage and complications that may occur in the implant, implant carrier and insertion spacers due to excessive torque during implantation [29].

There are many studies in the literature evaluating different surgical protocols for different bone densities. These studies have evaluated the effect of different protocols used during socket preparation, which is the first step of dental implant surgery, on implant IT [20,30,31]. However, no research has been identified on the effect of torque and insertion speed on implantation during the second stage of implantation. Although the companies’ socket preparation protocol is followed during implant surgery, extremely high torque values are achieved during implantation, especially in dense D1 bone, which prevents the implant from settling in the socket [32]. It can be seen that there is no optimal implantation protocol that allows the implant to be placed at the bone level in dense D1 bone. Establishing a protocol for dense D1 bone by determining the minimum insertion speed and torque values for implantation will minimise the complications that may occur with the bone and implant. The aim of this study was to evaluate the effect of two different insertion speeds at eight different ITs ranging from 25 to 60 during implantation in a dense PU D1 bone model on the placement condition and removal torque (RT) of dental implants.

## 2. Materials and Methods

This single-blind in vitro study was conducted at the Department of Oral and Maxillofacial Surgery, Faculty of Dentistry, Van Yüzüncü Yıl University in March 2024. Two 25 × 15 × 2.5 cm^3^ PU plates (PURYAP Construction Chemicals and Machinery Industry Trade. Co., Ltd., İstanbul, Turkey) with a density of 50 per cubic foot (pcf) were prepared for the study to be used as a dense D1 bone model in vitro. The literature suggests that the density of PU corresponding to D1 bone is 30–40 pcf and that corresponding to cortical bone is 50 pcf [33,34], so in this study, we chose to use single-layer 50 pcf PU plates to create a dense D1 bone model. The plates were coded by the assistants with the numbers 1 and 2 and the letters a, b, c, d, e, f, g and h to blind the author surgeon who would perform the implantation, and only the assistants knew which letter and number belonged to which group. The preparation of the implant sockets in the plates was performed according to a protocol established by an independent surgeon outside the study. Implantation in the groups was performed by the same author surgeon. A 3.75 × 10 mm^2^ dental implant (MarsTM, Medigma Biomedical GmbH, Wehingen, Germany) was used in the study. The implant placement condition and the insertion and RTs of the implants in the groups were measured and recorded. Based on the results of a previous study, the sample size was determined with an alpha error of 0.05, an effect size of 0.45 and a power (1-beta) of 0.80. A minimum sample size of 10 implant sockets was calculated for each subgroup [35].

### 2.1. Study Groups

**Figure 1:**
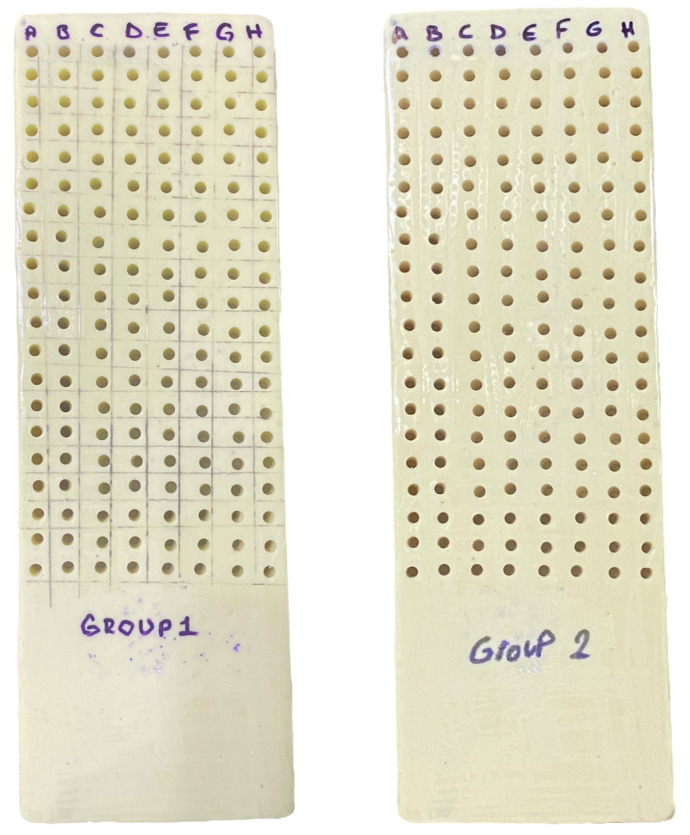
Preparation of implant sockets for Groups 1 and 2 (A = 25 torque group; B = 30 torque group; C = 35 torque group; D = 40 torque group; E = 45 torque group; F = 50 torque group; G = 55 torque group; F = 60 torque group).

In the study, a total of 320 implant sockets were divided into 2 groups according to insertion speed: 30 revolutions per minute (rpm) (group 1) and 50 rpm (group 2). Group 1 and Group 2 were divided into 8 subgroups as 25, 30, 35, 40, 45, 50, 55 and 60 torques. There were 20 implant sockets in each subgroup (Figure 1).

### 2.2. Implant Socket Preparation Protocol

The standard drilling protocol for 3.75 × 10 mm^2^ implants from the implant company was used to prepare the implant sockets in 8 subgroups in PU plates. Drilling was performed under saline irrigation using a physiodispenser at 1000 rpm with a torque setting of 70 and an implant handpiece with a 1:20 reduction. In Group 1, the implantation procedures were performed with the physiodispenser device in 8 subgroups of 25, 30, 35, 40, 45, 45, 50, 55 and 60 torque groups with the torque value of the respective group and a standard speed of 30 rpm. In Group 2, the implantation procedures were performed with the physiodispenser device in 8 subgroups of 25, 30, 35, 40, 45, 50, 55 and 60 torque groups with the torque value of the respective group and a standard speed of 50 rpm. During the implantations, the implant placement condition and RT values were assessed. The implant placement condition was evaluated as placement or non-placement of the implant at the bone level in the socket. For the RT value, the IT value of the corresponding group was taken as the reference, and the first value at which the implant rotated in the socket was taken as the RT, starting from the reference torque value and increasing by 5 torques in each trial. The RT was performed at the same rpm at which the implant was placed (implant removal was performed at 30 rpm for the 30 rpm group and 50 rpm for the 50 rpm group). The physiodispenser and implant handpiece could measure up to a maximum torque value of 80. For implants that could not be removed with a torque value of 80, a manual torque ratchet was used that could measure up to 100 torques. Implants with an RT value greater than 100 were considered to have an RT value of 100. In both groups, the distances between the implant sockets of the 8 subgroups were kept similar, and there were no complications in the implant sockets.

### 2.3. Statistical Analyses

SPSS 26.0.0 (Statistical Package for the Social Sciences) was used for statistical analyses in the evaluation of the results obtained in the study. In evaluating the study data, quantitative variables were presented using mean, standard deviation, median, minimum and maximum values, and qualitative variables were presented using descriptive statistical methods such as frequency and percentage. The Shapiro–Wilks test and box plots were used to assess the suitability of the data for normal distribution. Student’s *t*-test was used for quantitative evaluations of two groups showing normal distribution, a paired sample t-test for within-group evaluations, one-way ANOVA test for comparisons of three or more groups, and Bonferroni test to determine the group causing the difference. A chi-square test was used to compare qualitative data. Results were evaluated with 95% confidence interval and significance at the *p* < 0.05 level.

## 3. Results

A total of 320 implant sockets were included in the study. When the placement of the included implants in the sockets was analysed, it was found that 56.9% (n = 182) were placed in the socket at bone level and 43.1% (n = 138) were not placed in the socket. Implant RTs ranged from 40 to 100 with a mean of 80.81 ± 19.22 (Table 1).

There was a statistically significant difference between the 30 and 50 rpm groups in terms of overall implant placement condition (regardless of torque level) (*p* = 0.001; *p* < 0.01). When comparing the 8 subtorque groups, the rate of implant placement condition of the bone level was higher at 50 rpm than at 30 rpm at 35, 40 and 45 torque levels (*p* = 0.001; *p* < 0.01). There was no difference in the implant placement condition between 30 and 50 rpm at other torque levels (*p* > 0.05). When comparing the total implant RT values (regardless of torque level), it was found that the RT values at 50 rpm were statistically significantly higher than those at 30 rpm (*p* = 0.001; *p* < 0.01) (Table 2).

### 3.1. Intra-Group Implant Removal Torque Assessments

When the torque groups were evaluated in general and within the 30 and 50 rpm groups, a statistically significant difference was found between the implant RTs (*p* = 0.001; *p* < 0.01). Pairwise comparisons were made to determine the source of the difference; while the RTs of the implants in the 25 torque group were not significantly different from those in the 30 torque group (*p* > 0.05), they were lower than those in the 35, 40, 45, 50, 55 and 60 torque groups (*p* < 0.05). While the RTs of the implants in the 30 torque group were not significantly different from those in the 35 torque group (*p* > 0.05), they were lower than those in the 40, 45, 50, 55 and 60 torque groups (*p* < 0.05). While the RTs of the implants in the 35 torque group were not significantly different from those in the 40 torque group (*p* > 0.05), they were lower than those in the 45, 50, 55, 60 torque groups. The RTs of the implants in the 40 torque group were lower than those in the 45, 50, 55, 60 torque groups. The RTs of the implants in the 45 torque group were not significantly different from those in the 50, 55, 60 torque groups (*p* > 0.05). The RTs of the implants in the 50 torque group were not significantly different from those in the 55, 60 groups; the RTs of the implants in the 55 torque group were not significantly different from those in the 60 torque group (*p* > 0.05) (Table 3).

### 3.2. Inter-Group Implant Removal Torque Assessments

When the implant RT values were compared between the groups, it was found that the implant RTs at 50 rpm in the 25, 30, 35, 40, 50 and 55 torque groups were statistically significantly higher than those in the 30 rpm group (*p* = 0.001, *p* = 0.017; *p* < 0.01, *p* < 0.05). In the 45 and 60 torque groups, no statistically significant difference was found between the implant RTs at 30 and 50 rpm (*p* > 0.05) (Table 4).

## 4. Discussion

In recent years, PU has been reported to have similar biomechanical properties to human bone and also to have a more homogeneous average cell size than natural bone [23]. PU bone models are a standard and homogeneous material that can be produced in various thicknesses and densities from D1 to D4 [21,23]. Therefore, PU bone models are suitable for the biomechanical testing of dental implants such as IT, RT and resonance frequency analysis evaluation [20,21,34]. According to the Misch classification, a PU block with a density of 0.48–0.64 g/cc (30–40 pcf) resembles D1 bone in vivo and simulates cortical bone [36]. As reported in the literature, the PU block was preferred for modelling D1 bone in this study due to its many advantages. A 50 pcf PU plate was used to evaluate and model implant placement in dense D1 bone. There were no complications with the preparation of the implant sockets. Again, despite the high insertion and RTs during implant placement and removal, no complications were observed with either the plates or the implant sockets. The study used a physiodispenser and implant handpiece with a torque value of 80, which is the highest torque value of the devices currently used on the market and mimics the clinical environment [37]. In general, it can be seen that the RTs of implants with an IT of 45 and above are higher than 80. It is obvious that this situation, which we believe is due to both the dense D1 bone and the design of the implant. In cases where it is necessary to remove the implants in the clinical environment, it is obvious that the implant tools and handpieces used to remove the implant will wear out under these high torques, or the worn parts will not be able to remove the implant, causing unwanted complications [38,39]. When we evaluated the insertion speed, 80 and higher RT values were obtained at IT values of 35 and above in the 50 rpm group, and 80 and higher RT values were obtained at IT values of 45 and above in the 30 rpm group. Correspondingly, higher implant RTs were achieved in the 50 rpm group for implants with the same IT.

Implant stability is one of the most important factors for successful implant treatment, and primary implant stability is important for implant success and longevity [10]. Implant stability is essential for bone cell differentiation and osseointegration [40]. Satisfactory stability during the healing period prevents excessive micromovement and the disruption of bone formation [41]. Bone density, implant characteristics and surgical technique are the factors that influence primary implant stability [27]. The main characteristics of the implant are the implant material and the micro- and macro-design of the implant [42]. Grooves have been incorporated into implants to optimise the initial contact of the implant with the socket, increase stability [43], increase the surface area of the implant and positively distribute stress [44]. McCullough and Klokkevold reported that an aggressive groove design provides higher IT and primary stability [45]. Today, most implant manufacturers produce tapered implants. The reason for the preference for tapered implants is that tapered implants increase the primary implant stability by creating lateral compression in the bone in areas of weak bone and in cases with anatomical limitations [46]. Rokn et al. recommended the use of tapered implants to achieve better primary stability in areas of inadequate bone quality and quantity due to the greater lateral compression force that tapered implants exert on the surrounding bone [47]. Similarly, Lozano-Carrascal et al. reported that tapered implants had higher ISQ values and IT values than cylindrical implants [48]. In this study, we preferred a tapered implant in accordance with the literature. When comparing different bone types, the bone type with the highest primary implant stability is D1 bone. As bone density increases, primary stability also increases [13]. There is no study in the literature that investigates the minimum torque value at which the implant can be placed in different bone types. It can be seen that the focus of the studies is to determine the minimum torque suitable for immediate or early loading [49,50]. However, there are no data on the minimum torque at which the implant can be placed in the socket at bone level without being outside the socket in different bone types. High primary implant stability is desirable, but increasing torque is known to increase bone and implant-related complications [51]. In the search for the optimal implant IT that both allows early loading protocols and does not cause bone and implant-related complications, the optimal torque value should also meet the minimum torque value that allows the implant to be inserted at the bone level in different bone types. In this sense, this study was the first to evaluate the minimum implant IT at which the implant can be placed at the bone level in dense D1 bone type. In the 50 rpm group, the minimum torque level at which all implants were placed at the bone level was 40, whereas in the 30 rpm group, this value was set at 50 torque. In addition, while 80% of the implants in the 50 rpm group were placed at a torque value of 35, none of the implants in the 30 rpm group could be placed at the bone level. The fact that the RTs at which implants were placed at bone level in both groups were 80 and above is further evidence that these minimum values provide adequate primary stability.

In their study, which is the only study in the literature to evaluate implant insertion speed during implant placement, Hsu et al. found that implant stability, i.e., implant IT, decreased with increasing implant insertion speed in both good and poor bone types. In contrast to the study by Hsu et al., this study found that the rate of implant placement in the socket at the bone level and the implant RT increased with increasing insertion speed during implant placement [52]. We believe that the reason we found a different result from the study by Hsu et al. [52] is that the torque values were held constant when evaluating the effect of insertion speed in this study. Our results show that increasing the insertion speed at torque levels where the implant cannot be embedded in the socket, especially within certain limits, allows the implant to be placed at the bone level. The dense D1 bone type is particularly common in the anterior mandible and in the atrophic mandible [53]. When working in these cases, it is important to remember that the bone present may be very dense D1 bone, and in this case, we recommend using the placement torque and speed values presented in this study. In the study, we found that the RT values were higher than the IT values in all torque groups and speeds. We believe that this is due to the fact that the structural changes in the socket walls are minimal even at high ITs, particularly due to the density of the PU sheets used. These results suggest that a 50 pcf PU sheet can be used to model dense D1 bone. The implant used in the study was a tapered implant with self-tapping threads. It is possible that the results would have been different if an implant with different characteristics had been used. Although a standardised protocol was used to prepare the implant sockets, there may have been minimal surgeon-induced variation in the width of the sockets. Although we took care to ensure that the distance between the sockets was the same when creating the implant sockets, any minimal variations or differences that may have occurred could have affected the results by affecting the thickness of the socket walls.

## 5. Conclusions

In conclusion, this study was the first in the literature to evaluate the effect of implant insertion speed on implant placement at the bone level at different implant insertion torques in the dense D1 bone type. The 50 rpm group was found to have a higher rate of implant placement at the bone level than the 30 rpm group. In the dense D1 bone type, the minimum parameters in which all implants could be placed at the bone level were determined to be 50 torque when working at 30 rpm and 40 torque when working at 50 rpm. These results obtained for the dense D1 bone type should be confirmed in further in vitro and clinical studies. We also recommend further studies to evaluate the effects of the factors investigated in this study in implants with different designs and different bone types.

## Figures and Tables

**Table 1:** Descriptive characteristics.

		n (%)
Implant placement condition	Bone level	182 (56.9)
	Not fully inserted	138 (43.1)
Implant removal torque	Mean ± Sd	80.81 ± 19.22
	(Min − Max)	(40–100)
Group	30 rpm	160 (50.0)
	50 rpm	160 (50.0)
Torque subgroup	25 torque	40 (12.5)
	30 torque	40 (12.5)
	35 torque	40 (12.5)
	40 torque	40 (12.5)
	45 torque	40 (12.5)
	50 torque	40 (12.5)
	55 torque	40 (12.5)
	60 torque	40 (12.5)

**Table 2:** Comparison of implant placement condition and implant removal torque values according to rpm groups.

		30 rpm (n = 160)	50 rpm (n = 160)	^a^ *p*
Total	Bone level	66 (41.3)	116 (72.5)	^a^ 0.001 *
	Not fully inserted	94 (58.8)	44 (27.5)	
25 torque	Bone level	-	-	-
	Not fully inserted	20 (100)	20 (100)	
30 torque	Bone level	-	-	-
	Not fully inserted	20 (100)	20 (100)	
35 torque	Bone level	0 (0)	16 (80.0)	^a^ 0.001 *
	Not fully inserted	20 (100)	4 (20.0)	
40 torque	Bone level	2 (10)	20 (100)	^a^ 0.001 *
	Not fully inserted	18 (90)	0 (0)	
45 torque	Bone level	4 (20)	20 (100)	^a^ 0.001 *
	Not fully inserted	16 (80)	0 (0)	
50 torque	Bone level	20 (100)	20 (100)	-
	Not fully inserted	-	-	
55 torque	Bone level	20 (100)	20 (100)	-
	Not fully inserted	-	-	
60 torque	Bone level	20 (100)	20 (100)	-
	Not fully inserted	-	-	
Implant removal torque	Mean ± Sd	75.31 ± 21.49	86.31 ± 14.75	^b^ 0.001 *
	(Min-Max)	(40–100)	(50–100)	

^a^ Pearson chi-square test. ^b^ Student’s *t*-test. * *p* < 0.01.

**Table 3:** Intra-group comparison of implant removal torque values in rpm groups.

		Torque Subgroup		Mean ± Sd		(Min − Max)			^a^ *p*
Total (n = 320)		25 torque		56.00 ± 9.14		(40–70)			^a^ 0.001 *
		30 torque		61.50 ± 11.67		(40–80)			
		35 torque		70.50 ± 12.50		(45–100)			
		40 torque		76.00 ± 17.44		(45–100)			
		45 torque		92.00 ± 10.55		(75–100)			
		50 torque		95.00 ± 8.77		(80–100)			
		55 torque		96.00 ± 8.10		(80–100)			
		60 torque		99.50 ± 3.16		(80–100)			
30 rpm (n = 160)		25 torque		49.25 ± 6.13		(40–60)			^a^ 0.001 *
		30 torque		52.50 ± 10.07		(40–80)			
		35 torque		61.00 ± 10.08		(45–80)			
		40 torque		65.25 ± 15.85		(45–100)			
		45 torque		92.50 ± 10.58		(75–100)			
		50 torque		90.00 ± 10.26		(80–100)			
		55 torque		93.00 ± 9.79		(80–100)			
		60 torque		99.00 ± 4.47		(80–100)			
50 rpm (n = 160)		25 torque		62.75 ± 6.17		(50–70)			^a^ 0.001 *
		30 torque		70.50 ± 2.76		(65–75)			
		35 torque		80.00 ± 5.38		(70–100)			
		40 torque		86.75 ± 11.39		(70–100)			
		45 torque		91.50 ± 10.77		(75–100)			
		50 torque		100.00 ± 0.00		(100–100)			
		55 torque		99.00 ± 4.47		(80–100)			
		60 torque		100.00 ± 0.00		(100–100)			
Post hoc		25 torque	30 torque	35 torque	40 torque	45 torque	50 torque	55 torque	60 torque
Total	25 torque								
	30 torque	1.000							
	35 torque	0.001 *	1.000						
	40 torque	0.001 *	0.007 *	0.678					
	45 torque	0.001 *	0.001 *	0.001 *	0.004 *				
	50 torque	0.001 *	0.001 *	0.001 *	0.001 *	1.000			
	55 torque	0.001 *	0.001 *	0.001 *	0.001 *	1.000	1.000		
	60 torque	0.001 *	0.001 *	0.001 *	0.001 *	1.000	1.000	1.000	
30 rpm	25 torque								
	30 torque	1.000							
	35 torque	1.000	1.000						
	40 torque	0.372	1.000	1.000					
	45 torque	0.001 *	0.001 *	0.001 *	0.004 *				
	50 torque	0.001 *	0.001 *	0.001 *	0.012 *	1.000			
	55 torque	0.001 *	0.001 *	0.001 *	0.002 *	1.000	1.000		
	60 torque	0.001 *	0.001 *	0.001 *	0.001 *	1.000	1.000	1.000	
50 rpm	25 torque								
	30 torque	1.000							
	35 torque	0.006 *	0.347						
	40 torque	0.001 *	0.003 *	1.000					
	45 torque	0.001 *	0.001 *	0.490	1.000				
	50 torque	0.001 *	0.001 *	0.001 *	0.138	1.000			
	55 torque	0.001 *	0.001 *	0.002 *	0.256	1.000	1.000		
	60 torque	0.001 *	0.001 *	0.001 *	0.138	1.000	1.000	1.000	

^a^ One-way ANOVA test post hoc (Bonferroni test) * *p* < 0.01.

**Table 4:** Inter-group comparison of implant removal torque values in rpm groups.

Torque Subgroup	30 rpm (n = 160) Mean ± Sd (Min − Max)	50 rpm (n = 160) Mean ± Sd (Min − Max)	^a^ *p*
25 torque	49.25 ± 6.13	62.75 ± 6.17	0.001 *
	(40–60)	(50–70)	
30 torque	52.50 ± 10.07	70.50 ± 2.76	0.001 *
	(40–80)	(65–75)	
35 torque	61.00 ± 10.08	80.00 ± 5.38	0.001 *
	(45–80)	(70–100)	
40 torque	65.25 ± 15.85	86.75 ± 11.39	0.001 *
	(45–100)	(70–100)	
45 torque	92.50 ± 10.58	91.5 ± 10.77	0.769
	(75–100)	(75–100)	
50 torque	90.00 ± 10.26	100.00 ± 0.00	0.001 *
	(80–100)	(100–100)	
55 torque	93.00 ± 9.79	99.00 ± 4.47	0.017 *
	(80–100)	(80–100)	
60 torque	99.00 ± 4.47	100.00 ± 0.00	0.329
	(80–100)	(100–100)	

^a^ Student’s *t*-test. * *p* < 0.01.

## Data Availability

The raw data supporting the conclusions of this article will be made available by the authors on request.
